# Establishment of the Tree Shrew as an Alcohol-Induced Fatty Liver Model for the Study of Alcoholic Liver Diseases

**DOI:** 10.1371/journal.pone.0128253

**Published:** 2015-06-01

**Authors:** Huijie Xing, Kun Jia, Jun He, Changzheng Shi, Meixia Fang, Linliang Song, Pu Zhang, Yue Zhao, Jiangnan Fu, Shoujun Li

**Affiliations:** 1 College of Veterinary Medicine, South China Agricultural University, Guangzhou, Guangdong Province, PR China; 2 Guangdong Provincial Key Laboratory for the Prevention and Control of Severe Clinical Animal Diseases, Guangzhou, Guangdong Province, PR China; 3 Institute of Laboratory Animals, Jinan University, Guangzhou, Guangdong Province, PR China; 4 Medical Imaging Center, the First Attached Hospital of Jinan University, Guangzhou, Guangdong Province, PR China; University of Louisville School of Medicine, UNITED STATES

## Abstract

Currently, the pathogenesis of alcoholic liver diseases (ALDs) is not clear. As a result, there is no effective treatment for ALDs. One limitation is the lack of a suitable animal model for use in studying ALDs. The tree shrew is a lower primate animal, characterized by a high-alcohol diet. This work aimed to establish a fatty liver model using tree shrews and to assess the animals’ suitability for the study of ALDs. Tree shrews were treated with alcohol solutions (10% and 20%) for two weeks. Hemophysiology, blood alcohol concentrations (BACs), oxidative stress factors, alcohol metabolic enzymes and hepatic pathology were checked and assayed with an automatic biochemical analyzer, enzyme-linked immunosorbent assay (ELISA), western blot, hematoxylin-eosin (HE) staining and oil red O staining, and magnetic resonance imaging (MRI). Compared with the normal group, the levels of alanine aminotransferase (ALT), aspartate aminotransferase (AST), gamma-glutamyl transpeptidase (GGT), total cholesterol (TC), triglyceride (TG), reactive oxygen species (ROS), and malondialdehyde (MDA) were significantly enhanced in alcohol-treated tree shrews. However, the activity of reduced glutathione hormone (GSH) and superoxide dismutase (SOD) declined. Notable changes in alcohol dehydrogenase(ADH1), aldehyde dehydrogenase(ALDH2), CYP2E1, UDP-glucuronosyl transferase 1A1 (UGT1A1) and nuclear factor erythroid-related factor 2 (Nrf2) were observed. HE and oil red O staining showed that hepatocyte swelling, hydropic degeneration, and adipohepatic syndrome occurred in the tree shrews. Alcohol can induce fatty liver-like pathological changes and result in alterations in liver function, oxidative stress factors, alcohol metabolism enzymes and Nrf2. Therefore, the established fatty liver model of tree shrews induced by alcohol should be a promising tool for the study of ALDs.

## Introduction

Alcohol abuse has become a serious social problem. Overdrinking is associated with various liver diseases, resulting in a high incidence of disease and mortality due to liver injury [1]. Alcoholic liver diseases (ALDs) are caused by long-term excessive drinking. Initially, ALDs present as alcoholic fatty liver disease (AFLD). With continued drinking by the individual, AFLD can progress to alcohol steatohepatitis, fibrosis, cirrhosis, and, finally, to hepatocellular carcinoma [2]. AFLD is the first stage of ALDs and plays an important role in the occurrence and development of ALDs. The evolution of ALDs toward hepatitis, cirrhosis and liver cancer can be effectively prevented if a suitable treatment is undertaken at this stage of AFLD [3]. Although much has been reported on AFLD, the underlying pathogenesis is still not clear.

Oxidative stress refers to an increase in intracellular pro-oxidant species (i.e., reactive oxygen species, ROS). Excess ROS can cause mitochondrial damage, lipid peroxidation, DNA modification, etc., thereby resulting in tissue impairment [4]. Alcohols absorbed by the body are mainly metabolized by alcohol dehydrogenase (ADH), aldehyde dehydrogenase (ALDH), CYP450, and hydrogen peroxide enzymes [5]. A small portion of alcohol can be catabolized by UDP-glucuronosyltransferases (UGTs) [6]. The process of alcohol oxidation by various metabolic enzymes is accompanied with the production of a large quantity of reactive oxidative species (ROS) [7]. These ROS promote the formation of lipid peroxidation products (e.g., malondialdehyde), resulting in an imbalance of redox homeostasis [8]. This imbalance further reduces the levels of antioxidant species, such as superoxide dismutase (SOD) and glutathione (GSH), thus leading to structural and functional alterations of the hepatocytes [9, 10]. It is important to the pathogenesis of ALDs [11].

Nuclear factor erythroid-related factor 2 (Nrf2) is a series of essential transcription factors of antidotal and antioxidative genes. Nrf2 can facilitate high expression of multiple phase II enzymes that perform antioxidation and detoxification, which is one of the pivotal routes of the cellular antioxidant response [12, 13]. UGTs serve as important phase II antidotal enzymes and the downstream target genes can be regulated by Nrf2 [14]. UGTs can facilitate the binding of uridine diphosphoglucuronic acid to alcohols, which is followed by the alcohols being catalyzed into ethylglucuronide [15]. The liver is full of UGTs and is active in the metabolism of alcohol by glucuronide. Therefore, an appropriate liver model for monitoring the metabolism enzymes of alcohol is useful to an understanding of the pathogenesis of ALDs.

Human beings are a unique species in their voluntary oral alcohol uptake. However, using human subjects to study ALDs is ethically limited. Presently, research on the pathogenesis, prevention and treatment of ALDs is inevitably dependent on an animal model, *ex vivo* tissues, and cellular techniques [16]. Due to the heterogeneity of animals and the functional deficiency of cellular enzymes, the results based on current models have failed to clearly elucidate the pathogenesis of ALDs [17, 18]. It may be more suitable to use consanguine animals, such as primates, for the study of human ALDs. Tree shrews consume the flower buds of the bertam palm daily for food; the nectar in these buds is approximately 3.8% alcohol [19]. The Chinese tree shrew (*Tupaia belangeri chinensis*) possesses many features similar to those of humans and is frequently used as an experimental model in biomedical research [20]. However, the potential use of the Chinese tree shrew as an alcohol-induced fatty liver model animal has not been explored.

In this study, a fatty liver model was established using Chinese tree shrews that were allowed to drink alcohol. Various biochemical indexes, oxidative stress factors, protein expression of principal alcohol metabolic enzymes and Nrf2, and liver histopathology of the tree shrews were checked after the alcohol treatment. We evaluated the feasibility of an AFLD model induced by feeding alcohol to the tree shrews. Finally, the effects of alcohol induction on the pathogenesis of AFLD were discussed, based on analyses of liver function, oxidative stress, protein expression of enzymes and Nrf2, and histopathology.

## Materials and Methods

### Materials

Edible alcohol was purchased from Beijing Red Star Co., Ltd (Beijing, China). Test kits for ROS, SOD, GSH and MDA were provided by Nanjing Jiancheng Bioengineering Institute (Nanjing, China). Antibodies against ADH1, ALDH2, CYP2E1, UGT1A1, Nrf2 and goat anti-rabbit IgG-HRP were obtained from Santa Cruz Biotechnology (Dallas, TX, USA). HRP-conjugated anti-β actin monoclonal antibody (1C7) was acquired from Beyotime Institute of Biotechnology (Shanghai, China). Oil red O and HE staining kits were purchased from Sigma-Aldrich (St. Louis, MO, USA). A human saliva peroxidase (SP) ELISA and DAB staining kits were supplied by CUSABIO Biotechnology Co., Ltd (Wuhan, China).

### Subjects and Alcohol Feeding

Male tree shrews (120 g ± 10 g) were purchased from the experimental animal center of Kunming Medical College (Kunming, China). The animals were housed in an alternate light-dark cycle (12 h) room with a temperature of 22 ± 2°C and a relative humidity of 50–60%. Tree shrews were fed with a complete formula food and allowed water ad libitum. The tree shrews underwent a one-week adaption period before the experiment.

Animal experiments were in accordance with the Guidelines on the Care and Use of Animals for Scientific Purposes (2004, Singapore). The protocols for the animal studies were also reviewed and approved by the Experimental Animal Ethical Committee of Jinan University (Certification No: 20140301). Tree shrews were randomly divided into control, 10% and 20% alcohol (v/v) groups (*n* = 6). The control group was allowed free access to food and water. The alcohol groups were given a 5% alcohol solution instead of water on the first day. Afterwards, the alcohol increased every other day along a 5% gradient until the alcohol content reached the predetermined levels. Alcohol feeding proceeded for 14 days after the achievement of the fixed alcohol levels. During the experiment, the general conditions such as mental state, appetite and body weight of experimental animals were investigated and recorded.

### Biochemical Assays

Blood samples were collected from the posterior vena cava of the tree shrews on the last day of the experiment. After standing for 10 min, the samples were centrifuged at 3000 rpm for 10 min to separate the serum. Subsequently, the serum samples were subjected to assays for alanine aminotransferase (ALT), aspartate aminotransferase (AST), gamma-glutamyl transpeptidase (GGT), total cholesterol (TC), and total triglycerides (TG) using a biochemical analyzer (COBAs Integra 400 Plus, Roech, Basel, SUI) or commercially available enzymatic assay kits. The BACs of tree threws were determined at the last day by EnzyChrom ethanol assay kit following the protocol recommended by the manufacturer (Bioassay Systems, Hayward, CA). Each assay was performed in triplicate.

### Determination of Oxidative Stress Factors

Serum and liver samples were assayed for MDA, ROS, SOD and GSH according to the manufacturer’s instructions. All quantifications for oxidative stress factors were repeated three times with the data shown as the mean ± SD.

### Western Blot Analysis of Alcohol Metabolic Enzymes

Liver tissues were sampled from the tree shrews after the animals were sacrificed by CO_2_ asphyxiation. The samples (100 mg) were homogenized in 1 mL 1× RIPA buffer (Radioimmune precipitation buffer containing protease inhibitor cocktail set I, Beyotime, Shanghai, China) after thawing. Then, the liver lysates were centrifuged at 14,000 rpm for 10 min at 4°C and protein concentrations were determined by the BCA assay for immunoblotting. After separation using SDS-PAGE, the protein expressions of various alcohol-associated enzymes and Nrf2 were detected based on western blot. PVDF membrane was used for the electrophoretic transfer of protein from the gel (Haoran Biotech, Shanghai, China). The membrane was first blocked with 5% fat-free milk for 1 h at room temperature and then incubated with primary antibodies against ADH1 (1:2000), ALDH2(1:1000), CYP2E1(1:5000), UGT1A1(1:2000), and Nrf2 (1:1000). The signals were detected by using HRP-conjugated rabbit anti-goat (1:1500) and anti-β actin monoclonal (1:800) secondary antibodies. NIH-Image software was used to quantify the optical density of protein bands with the value of the β actin band as a reference.

### Histological Examination of Liver Tissue

HE staining and oil red O staining were adopted to examine the histological alterations of liver tissue. Briefly, liver tissues preserved in 4% paraformaldehyde were dehydrated using Carnoy's fluid and then used to prepare paraffin-embedded sections. After being stained by HE and oil red O, the liver histomorphology of the tree shrews was observed and photographed using an Olympus imaging system. The hepatic pathology was scored according to the severity of liver injury using HE and oil red O staining sections.

To further investigate the occurrence of AFLD, MRI was adopted to scan the liver after the animals were rendered unconscious by injection of Ketamine Hydrochloride (40mg/kg). MRI was performed on a GE Signa magnetic resonance spectrometry (1.5 T) using T2-weighted imaging(T2WI) and T2WI fat-suppression(fs) scanning sequences.

### Statistical Analysis

The resulting data are shown as the mean ± SD (*n* = 6). An analysis of variance (ANOVA) was performed to determine significance using SPSS 16.0 (SPSS, NY, USA) if the variance between the two groups was homogenous; otherwise, a nonparametric test was applied. A *P* value < 0.05 was considered significant.

## Results

### General conditions

**Table 1**shows the general conditions of tree shrews before and after alcohol feeding for 14 days. The tree threws of control were facile and active in action, and kept a continuous increase of body weight. Also, there was no abnormal changes of liver in terms of anatomic check. However, alcohol-treated groups appeared loss of appetite, mental suppression, decreased mobility, and body weight loss to some extent. Liver dissection exhibited yellow texture and hepatomegaly, and the 20% alcohol-treated group was more significant in pathological changes.

**Table 1:** General conditions of tree shrews before and after feeding with alcohol solutions for 14 days.

Group	Body weight (g)		Appetite/faeces		Mental state	
	0 d	14 d	0 d	14 d	0 d	14 d
Control	123.6±3.5	128.6±4.9	Normal	As before	Normal	As before
10% alcohol	123.7±5.6	101.9±4.8*	Normal	Appetite loss (+) /brown faces	Normal	Mobility (-); Depression (+)
20% alcohol	126.1±6.3	87.9±7.3†	Normal	Appetite loss (++) /glutinous faces	Normal	Mobility (--); Depression (++)

*Pared-t test, *significantly different from the control (*p*<0.01)

†significantly different from the 10% alcohol group (*p*<0.01).

+ denotes the extent of severity positively, and - denotes reversely.

### Blood Biochemical Indices

Compared to the control group, the serum ALT, AST, GGT, TC and TG levels of the alcohol-treated groups significantly increased (**Table 2**). Relative to the 10% alcohol-treated group, the 20% alcohol group possessed higher liver enzyme levels, indicating an elevated activity of alcohol metabolism. Markedly increased TC and TG were also discovered in the alcohol-treated tree shrews. Moreover, there was a significant difference in the ALT (*P* < 0.01), GGT (*P* < 0.01) and TC (*P* < 0.05) between the 10% and 20% alcohol groups.

**Table 2:** Blood biochemical indices of tree shrews after different treatments for 14 days. Data shown as the mean ± SD (*n* = 6).

Group	ALT (IU/L)	AST (IU/L)	GGT (mmol/L)	TC (mmol/L)	TG (mmol/L)
Control	44.17±16.01	159.50±17.67	1.67±0.81	2.25±0.39	1.06±0.08
10% alcohol	69.83±23.28*	593.84±222.52*	4.17±0.98*	3.26±0.81*	1.45±0.48*
20% alcohol	157.67±41.85*,†	619.33±283.53*	43.5±27.71*,†	4.92±1.40*,†	1.35±0.21*

ALT: alanine aminotransferase; AST: aspartate aminotransferase; GGT: gamma-glutamyl transpeptidase; TC: total cholesterol; TG: total triglycerides.

One-way ANOVA, *significantly different from the control (*p*<0.01)

†significantly different from the 10% alcohol group (*p*<0.01)

The BAC of control group was 24.54 ± 8.11 mg/dL. The BACs of alcohol-treated tree shrews were up to 45.47 ± 3.92 mg/dL (10% alcohol group) and 69.28±9.07 mg/dL (20% alcohol group), respectively. The BACs of tree shrews were significantly enhanced after alcohol feeding in comparison with the normal group (*P*<0.01), which was more conspicuous for high-alcohol treated tree shrews.

### Oxidative Stress Factors

The levels of oxidative stress factors of the tree shrews after being fed alcohol solution for 14 days are shown in **Table 3**. In comparison with the control group, the ROS and MDA levels both in the blood and liver of the alcohol-treated groups were significantly elevated. Conversely, the levels of GSH and SOD markedly declined. The changes in the oxidative stress factors were alcohol concentration-dependent. High alcoholic feeding resulted in a more significant elevation or decline in the index factors. Differences in various factors between the 10% and 20% alcohol groups were statistically significant (*P*<0.05). Increased oxidative products and decreased reductive species indicated that the oxidative stress injury occurred in the alcohol-treated tree shrews.

**Table 3:** Changes of oxidative stress factors in the blood and liver of tree shrews after feeding with water or alcohol solution.

Group	ROS		MDA		GSH		SOD	
	Serum (nmol/mL)	Liver (ng/mgprot)	Serum (nmol/mL)	Liver (ng/mgprot)	Serum (nmol/mL)	Liver (ng/mgprot)	Serum (nmol/mL)	Liver (ng/mgprot)
Control	540.25±46.32	1.63±0.06	0.31±0.12	0.09±0.01	602.74±76.78	1.20±0.16	662.50±50.57	21.89±1.48
10% alcohol	635.08±62.43	1.72±0.06	0.62±0.10*	0.14±0.02	472.22±50.62*	1.38±0.14	450.63±49.34*	19.19±1.27
20% alcohol	698.36±56.19*	2.04±0.13*	1.05±0.23*	0.19±0.04*	387.85±30.05*	0.89±0.15*	367.30±39.90*	15.96±1.18*

Pared-t test, *significantly different from the control, *p*<0.05.

### Expression of Alcohol Metabolic Enzymes and Nrf2

**Fig 1:**
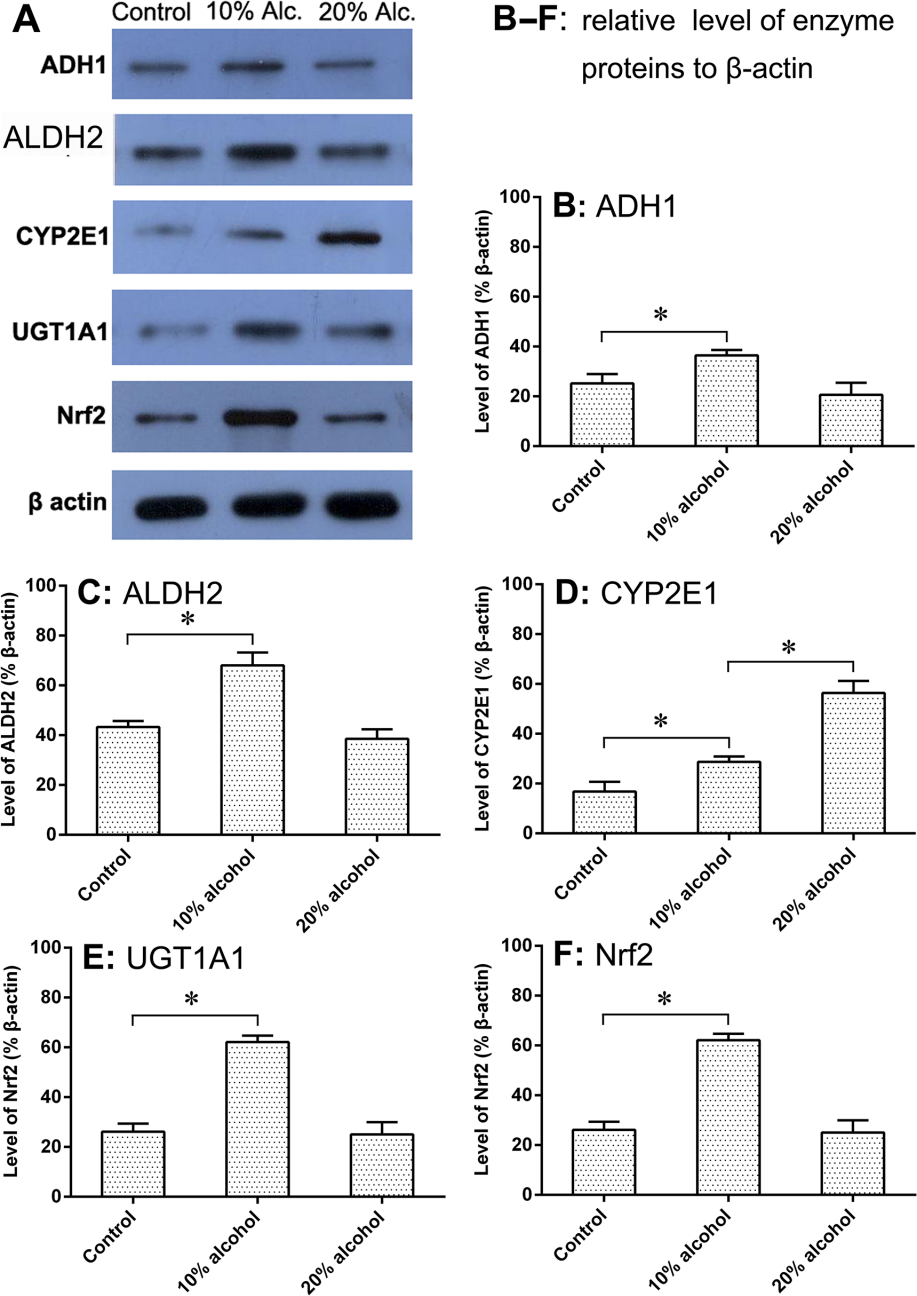
Levels of alcohol metabolic enzymes in the liver of tree shrews analyzed by western blot after treatment with water or alcohol solutions for 14 days. Feeding alcohol can cause significant changes in the level of various alcohol metabolic enzymes. (**A**) The protein bands of the alcohol metabolic enzymes detected by western blot; (**B–F**) Protein expressions of ADH1, ALDH2, CYP2E1, UGT1A1 and Nrf2 relative to the internal reference protein β-actin with no, low, and high levels of alcohol treatment. All data are expressed as the mean ± SD, an asterisk (*) denotes a significant difference between one another with p < 0.05.

The expressions of the various alcohol metabolic enzymes and the Nrf2 of tree shrews were assayed by protein immunoblot. **Fig 1**shows the results of the western blot (**A**) and the relative expression of the proteins related to alcohol metabolism (**B-F**). Compared to the control group, the expression of ADH1 significantly increased in the 10% alcohol group, whereas the protein expression varied less when subjected to the 20% alcoholic feeding. There was also no significant difference (*P* >0.05) between the control group and the 20% alcohol group in ADH1 expression. The expression of ALDH2 showed a similar trend to ADH1, characterized by ascent at the lower alcohol level and balance at the higher alcohol level. The expression of CYP2E1, in contrast, always increased with the enhancement of alcohol concentration. The change in the CYP2E1 level was statistically significant among groups (*p* <0.05). Levels of UGT1A1 and Nrf2 were consistent with levels of ADH1 and ALDH2 in the protein expression. All alcohol metabolic enzymes and Nrf2 increased in the tree shrews that were treated with a low-level alcohol solution (10%). However, the tree shrews given the 20% alcohol beverage showed enzyme levels (except for CYP2E1) and Nrf2 levels parallel with the control group. The capacity of CYP2E1 to metabolize alcohol was positively correlated with alcohol concentration, which accorded with Lu’s report [10].

### Histomorphology of Liver Tissues

**Fig 2:**
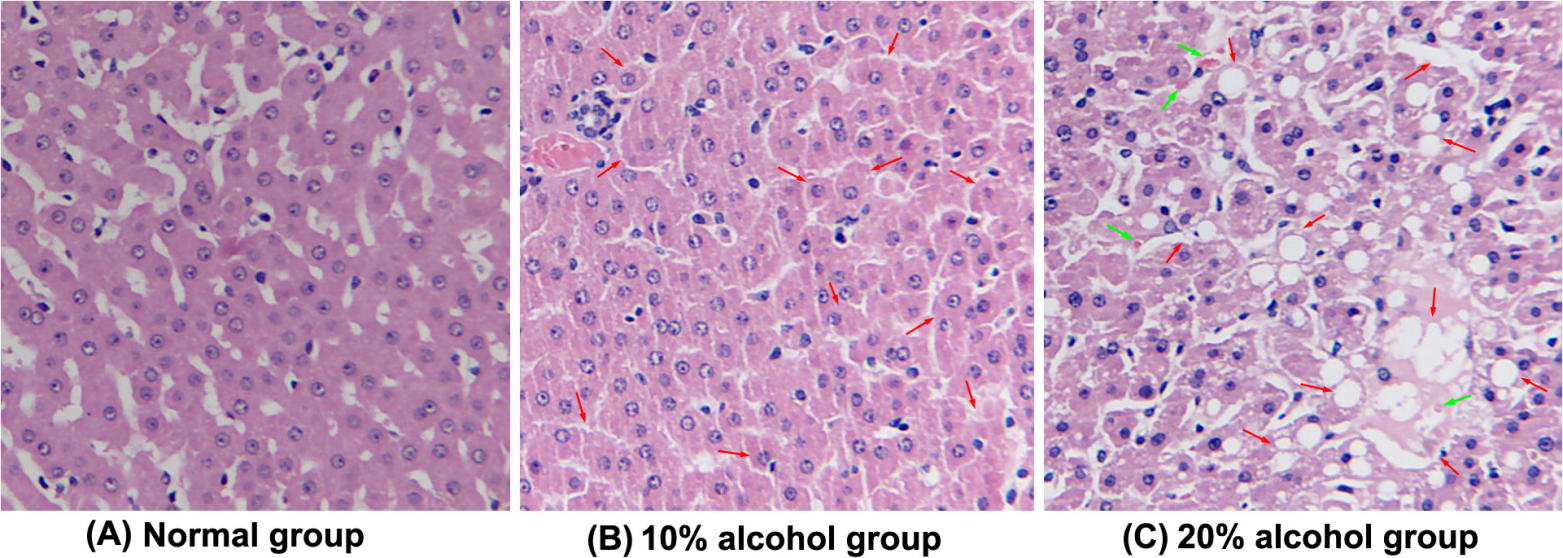
Representative histological feature of liver sections stained with HE (200×), depicting the effect of different treatments on the liver histopathology of tree shrews with AFLD. (**A**) Normal control group; (**B**) 10% alcohol-induced fatty liver with macrovesicles (indicated by arrowhead) of hepatocytes was graded as moderate in the AFLD group; (**C**) 20% alcohol-induced fatty liver with macrovesicles (indicated by red arrow) of hepatocytes was graded as high in the AFLD group. The green arrow represents the formation of Mallory bodies.

The HE staining results of the liver sections are presented in **Fig 2**. The tree shrews receiving the regular diet had normal liver histomorphology. However, the 10% alcohol group exhibited obvious pathological changes, including swelling of the hepatocytes, disarrangement of cell cords, etc. The pathological changes of the liver were more significant for the 20% alcohol-treated tree shrews. Hydropic degeneration, disarranged cell cords, endolysis, formation of Mallory bodies, and inflammatory cell infiltration were found in the HE-stained liver sections. HE staining suggested that alcohol feeding can result in the formation of ALDs in tree shrews.

**Fig 3:**
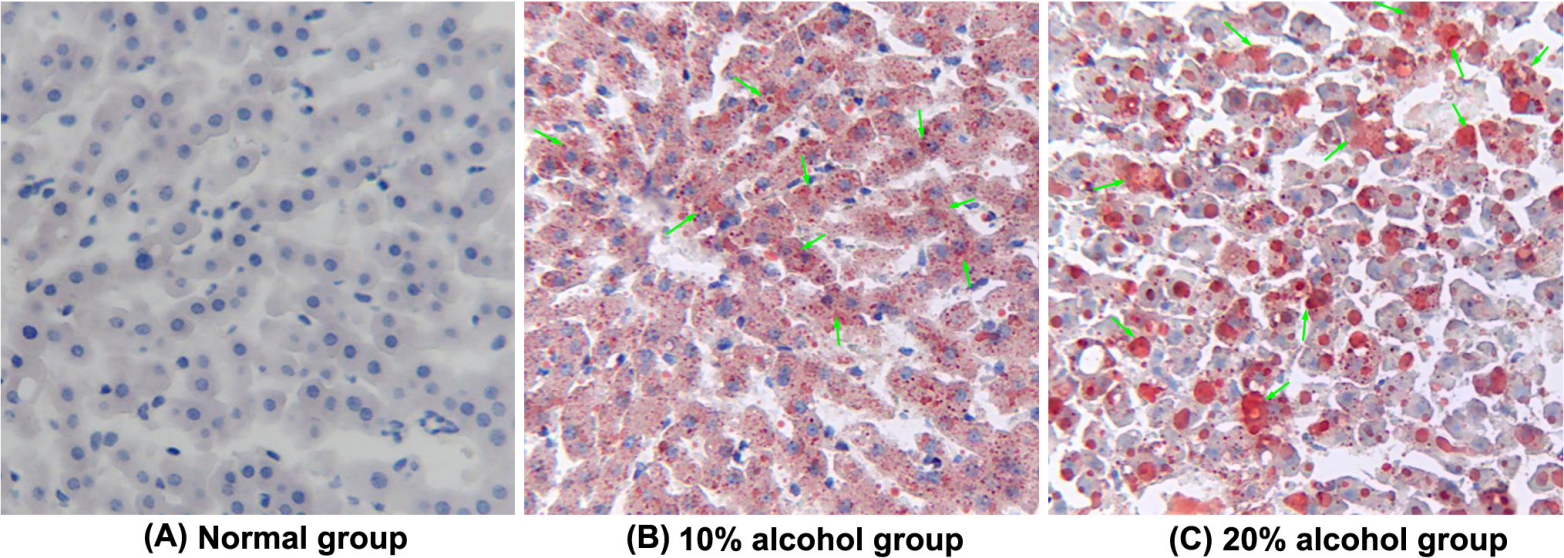
Representative histological feature of liver sections stained with oil red O staining (200×), depicting the effect of different treatments on the liver histopathology of tree shrews with AFLD. (**A**) Normal control group. (**B**) 10% alcohol-induced fatty liver with fat vacuoles (indicated by arrowhead) around the hepatocytes was graded as moderate in the AFLD group. (**C**) 20% alcohol-induced fatty liver with fat vacuoles (indicated by arrowhead) around the hepatocytes was graded as high in the AFLD group.

For further detection on the progress of fat accumulation, oil red O was applied to stain the liver sections. **Fig 3**displays the typical histomorphology of liver tissues of tree shrews after 14 days treatment. There were no obvious fatty droplets found in the micrograph of the liver section of the normal tree shrews. For alcohol-treated groups, red fat vacuoles stained by oil red O were clearly presented on the micrographs, of which, the fatty staining in the 20% alcohol group was more evident. It was an alcohol concentration-dependent fat accumulation process. With the increase of alcohol level, the adipohepatic degree (red) of the liver appeared more and more prominent, showing a diffuse fatty infiltration of the liver. Generally, ALDs can be divided into alcoholic mild liver (AML), alcoholic fatty liver (AFL), alcoholic hepatitis (AH), alcoholic liver fibrosis (ALF), and alcoholic liver cirrhosis (ALC) according to the severity of liver injury. In the case of tree shrews, there was no fibrous degeneration of hepatocytes observable. Taken HE and oil red O micrograph together, the liver injury of tree shrews has reached the grade of AH. The results revealed that alcohol feeding can induce a syndrome of AFLD in tree shrews, especially at a high alcohol level.

**Fig 4:**
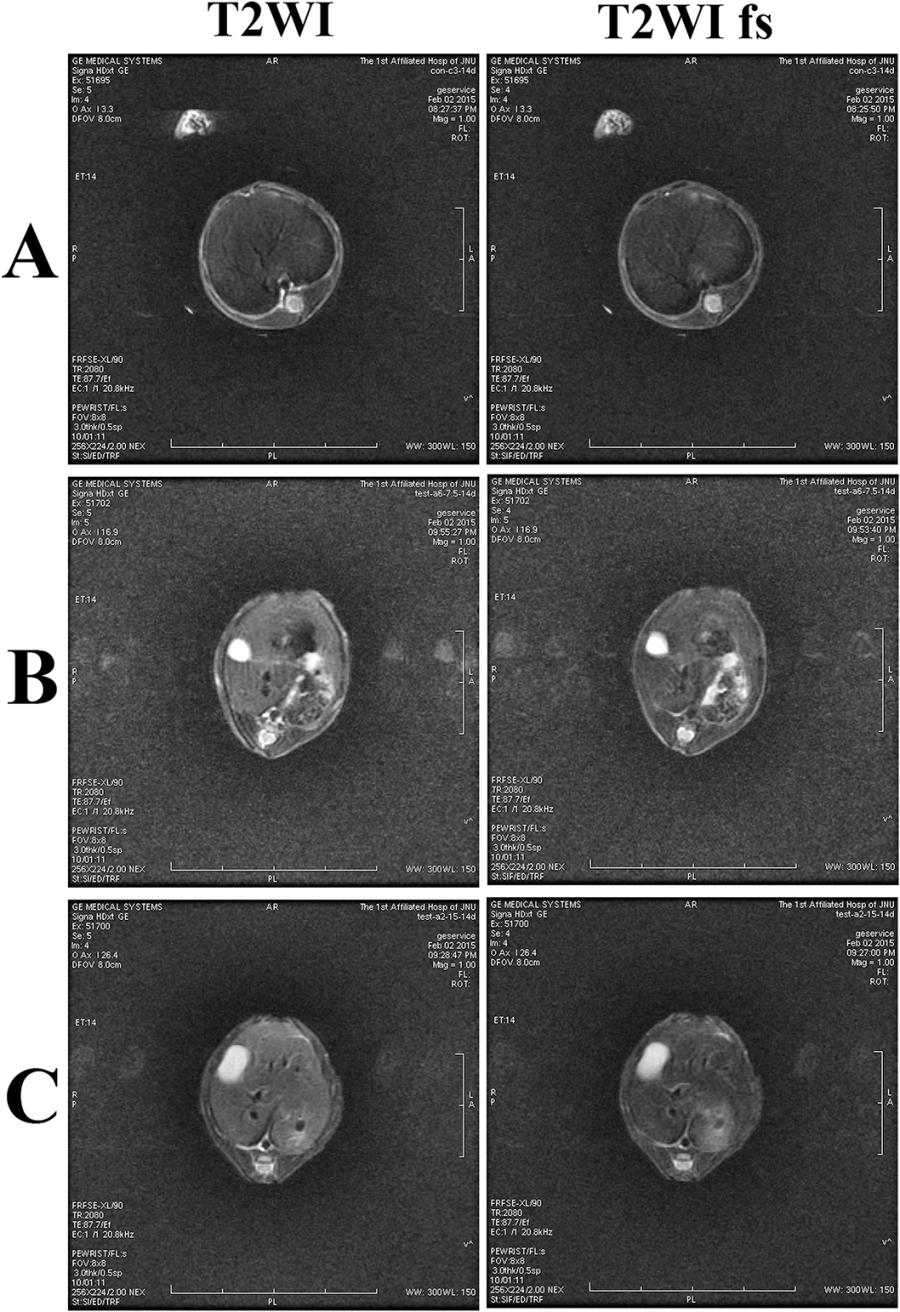
Liver MRI images of the control and experimental tree shrews after 14 days through T2WI andT2WI fs scanning: (A) control group, (B) 10% alcohol group, and (C) 20% alcohol group. MRI indicated that alcohol feeding can induce the development of AFLD.

MRI check also indicated a formation of AFLD in tree shrews. From the MRI images (**Fig 4**), it could be seen that the alcohol-treated groups exhibited higher liver signals than the control group. The signal intensity of high-alcohol group was also slightly higher than that of the low-alcohol group. By comparing T2WI and T2WI fs signals, there was no significant difference after fat suppression. However, the signals increased after fat suppression sorting as far as 10% and 20% alcohol-treated groups concerned. The results of MRI indicated alcohol feeding can induce the development of AFLD of tree shrews.

## Discussion

The principal metabolism of alcohol (~90%) occurs in the liver, and the liver easily injures after heavy drinking. This leads to the increase of hepatocellular permeability due to cell stress or impairment. ALT and AST mainly exist in the mitochondria of hepatocytes that are rapidly released into the blood when the liver injury is induced by alcohol, thereby resulting in the enhancement of ALT and AST levels. The Lieber-Decarli alcohol diet is the commonly used fatty liver-induced model [21]. However, only mild steatosis and ALT elevation can be obtained in mice with a continuous Lieber-Decarli alcohol diet for 4 weeks [22, 23]. The induction of AFLD needed a 12-week intervention, but the ALT level was only moderately elevated [24]. Thus, the AFLD model based on mouse or rat is limited for the study of the pathogenesis of alcohol-related diseases. Tree shrew is characterized by higher base serum ALT and AST levels than other species, perhaps making them more suitable to construct AFLD model. When tree shrews were given alcohol solutions for 2 weeks, the levels of ALT, AST, GGT and TC were significantly enhanced. The elevation of liver indices was related to the alcohol concentration, with the higher alcohol solution (20%) resulting in higher scores. Biochemical assays indicated that the tree shrew was more suitable for the establishment of an AFLD model.

Presently, oxidative stress and lipid peroxidation are considered responsible for AFLD [25]. The metabolism of alcohol by ADH, ALDH and CYP2E1 produces a lot of acetaldehydes that have direct effects on hepatocyte injury. Additionally, acetaldehyde can induce excessive ROS to mediate the process of lipid peroxidation, leading to the formation of considerable MDA [26]. The level of *in vivo* MDA is an indicator of lipid peroxidation in the body, which can reflect the extent of oxidative stress injury caused by an attack of free radicals [27]. In our study, it was found that the ROS and MDA levels increased in tree shrews when they were exposed to alcohol. Regarding oxidative stress, excessive ROS and MDA will consume a large quantity of antioxidation factors, such as SOD and GSH. Once the balance is lost, the *in vivo* SOD and GSH would be unable to fight against the excessively increased ROS and MDA. Maintaining suitable levels of SOD and GSH plays an important role in the protection of the liver from attacks of free radicals [28]. Our results show that the levels of SOD and GSH in the blood of the tree shrews declined significantly when the tree shrews were subjected to the alcohol diet. The changes in ROS, MDA, SOD and GSH were alcohol concentration-dependent. High alcohol feeding (20%) can cause a more significant oxidative stress.

Another concern is the change in alcohol metabolic enzymes and related regulatory factors when modeling the fatty liver. Approximately 90% of the alcohol absorbed into the body is oxidized in the liver by ADH, ALDH and CYP2E1; a small amount of the alcohol can be catalyzed by UGTs; and the rest is excreted through the spleen and kidney [29, 30]. Nrf2 is the key target for regulating oxidative stress [31]. Nrf2 is referred to as the master regulator of the antioxidant response that modulates the expression of various antioxidant enzymes [32]. Additionally, Nrf2 can upregulate the expression of UGT1A1, a phase II enzyme that has a higher metabolic activity in alcohol [33]. Thereby, Nrf2 can significantly reduce the alcohol-mediated oxidative stress injury of liver cells by reinforcing the effects of antioxidation and detoxification [33]. The western blot results showed that the enzyme expression, apart from CYP2E1, of the tree shrews in the 10% alcohol group increased significantly (*p* <0.05); however, the 20% alcohol group presented enzyme levels parallel to those of the normal group. The expression of CYP2E1 regularly increased with the enhancement of alcohol concentration, suggesting a difference in the metabolism capacity between CYP2E1 and the other enzymes. High expression of CYP2E1 may be an important cause of the development of alcohol-mediated AFLD. Likewise, the expression of the Nrf2 in the tree shrews displayed a similar trend to most of the alcohol metabolic enzymes. These results indicate that tree shrews possess a high alcohol tolerance, which is the reason why tree shrews are not intoxicated after the intake of alcohol equal to a toxic dose in humans. Tree shrews are closely related to primates and have been used as an alternative to primates in experimental studies [34]. In terms of our findings, there were some similarities in enzymes and Nrf2 changes between the tree shrews and human beings following the consumption of alcohol. This suggested to us that the AFLD model established with tree shrews will be of high value when used in the study of ALDs.

In order to verify the formation of AFLD, HE and oil red O staining were applied to investigate the liver histomorphology of tree shrews. The micrograph of the HE staining revealed that 10% alcohol induced the cytolysis of partial liver cells and the production of a small number of vacuoles. Compared to the 10% alcohol, the 20% alcohol resulted in severe hydropic degeneration and a large quantity of vacuolar cells. Similarly, oil red O staining demonstrated that alcohol feeding could cause the adipohepatic alteration of tree shrew liver, and the degree of adipohepatic alteration was more acute for the 20% alcohol group. Moreover, MRI check also demonstrated the occurrence of AFLD of tree shrews when the animals were given alcohol drinking for two weeks. Overall, alcohol feeding can induce a series of changes in the biochemical parameters, alcohol metabolic enzymes, and liver histomorphology of tree shrews that are similar to the syndrome of fatty liver. Our findings indicate that the establishment of a fatty liver model using tree shrews is feasible, which may be helpful to study, prevent, and cure ALDs.

## Conclusions

An alcohol-induced fatty liver model was established in this study using the tree shrew, a new experimental animal. Alcohol feeding can induce liver injury in the tree shrew via oxidative stress, along with elevated levels of ALT, AST, GGT, TC and TG. Low-level alcohol (10%) can induce a moderate fatty liver syndrome in tree shrews, with significantly elevated liver physiological indices. However, the fatty liver lesion can be easily obtained using a high level of alcohol (20%). Therefore, the fatty liver model established in tree shrews is highly flexible. It also provides a useful tool for studying the pathogenesis of ALDs.
